# Twisting two-dimensional iron sulfide layers into coincident site superlattices *via* intercalation chemistry†

**DOI:** 10.1039/d3sc02994h

**Published:** 2024-01-24

**Authors:** Lahari Balisetty, Brandon Wilfong, Xiuquan Zhou, Huafei Zheng, Sz-Chian Liou, Efrain E. Rodriguez

**Affiliations:** a Department of Chemistry and Biochemistry, University of Maryland College Park MD 20742 USA efrain@umd.edu; b Maryland Quantum Materials Center, University of Maryland College Park MD 20742 USA; c Electron Microscopy Facility, Institute for Functional Materials and Devices, Lehigh University Bethlehem PA 18015 USA

## Abstract

Layered van der Waals (vdW) materials are susceptible not only to various stacking polymorphs through translations but also twisted structures due to rotations between layers. Here, we study the influence of such layer-to-layer twisting through the intercalation of ethylenediamine (EDA) molecules into tetragonal iron sulfide (Mackinawite FeS). Selected area electron diffraction patterns of intercalated FeS display reflections corresponding to multiple square lattices with a fixed angle between them, contrary to a single square lattice seen in the unintercalated phase. The observed twist angles of 49.13° and 22.98° result from a superstructure formation well described by the coincident site lattice (CSL) theory. According to the CSL theory, these measured twist angles lead to the formation of larger coincident site supercells. We build these CSL models for FeS using crystallographic group-subgroup transformations and find simulated electron diffraction patterns from the model to agree with the experimentally measured data.

## Abstract 2

TEM Electron diffraction analysis of twisted coincident site superstructures in intercalated tetragonal iron sulfide.

## Graphical abstract

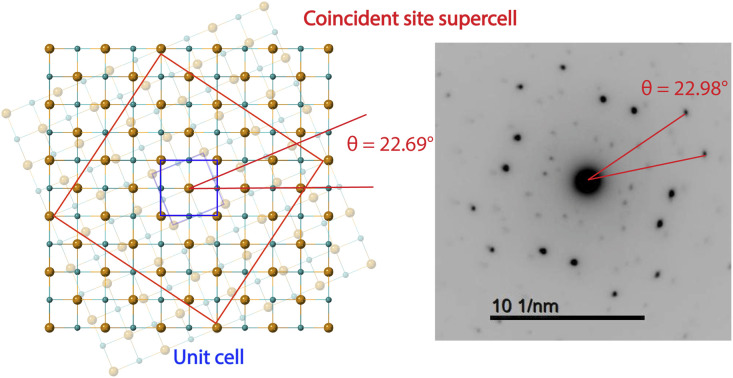

## Introduction

Two-dimensional (2D) layered materials are a versatile family of compounds capable of being formed into interesting architectures due to the van der Waals (vdW) gap along the stacking direction. Researchers have exploited this bonding characteristic to design certain structures, *e.g.*, heterolayers, for target applications.^1–3^ Here, we present a chemical means by which to twist vdW layers and focus on tetragonal iron sulfide, a 2D layered material with Fe atoms sandwiched between layers of chalcogenide (S^2−^) anions.^4–6^ This metastable anti-PbO structure type of iron sulfide (FeS) is also found in nature as the mineral mackinawite.^7–10^ FeS is metallic and displays interesting physics such as superconductivity, its critical temperature *T*_c_ being close to 5 K.^11^

We chose mackinawite FeS for this study since it expands the library of known vdW metal chalcogenides (*e.g.*, semiconducting transition metal dichalcogenides) and because it is known to host a variety of guest species. For example, one can insert into its interlayer galleries alkali metal ions,^12^ amines,^13^ metal amine complexes,^14–16^ and metal hydroxide layers.^17^ This intercalation chemistry goes beyond a synthetic interest too. In the isostructural tetragonal iron selenide phase, the *T*_c_ was found that intercalated samples were found to exhibit higher *T*_c_s than the parent compound. While *T*_c_ starts at 8 K in FeSe, in the intercalated phases it can reach up to 48 K.^18–28^ While such high temperatures have not been found in the sulfide analogue, Zhou *et al.* found that FeS could go as high as 8 K when cationic (Li_1−*x*_Fe_*x*_OH)^*δ*+^ layers grow within the FeS interlayer galleries.^17^ Regardless, these studies emphasize the important effects of intercalation chemistry on the electronic properties of vdW materials.

In addition to charge doping into the host electronic bands, intercalation species can have important consequences in the host's structure. Since bonding in layered FeS is anisotropic in nature with strong covalent bonding in the *ab*-plane and weak vdW interactions along the *c*-axis, the FeS structure possess two dominant degrees of freedom: an in-plane translation and an in-plane rotation. Furthermore, FeS crystallizes in a centrosymmetric *P*4/*nmm* space group with an inherent inversion center. Any structural manipulation such as changing the stacking sequence, alignment, or orientation of the 2D layers can break inversion symmetry and yield non-centrosymmetric structures. The transition metal dichalcogenide (TMD), MoS_2_, can exhibit symmetry breaking *via* stacking sequence (*i.e.*, translational symmetry). The thermodynamically stable and centrosymmetric 2H–MoS_2_ phase displays ABAB stacking while the exotic and non-centrosymmetric 3R-polymorph has ABCABC stacking.^29^ Loss of inversion symmetry in the 3R-phase allows for its study and application in non-linear optical devices.^30,31^ In the realm of quantum materials, the TMDs TiS_2_ and TaS_2_ have been intercalated with chiral methylbenzylamine molecules to induce spin selectivity without the use of an external magnetic field.^32^ Clearly, synergy between the intercalate and host structures opens new opportunities of physical properties in vdW materials.

Another approach to removing the inversion center is through in-plane twisting of the 2D sheets. This recent branch of research named twistronics,^33,34^ has garnered attention because of the report on unconventional superconductivity in graphene observed due to twisting of the layers by an angle of exactly 1.1 degrees.^35,36^ The twisted 2D sheets problem is being extended to observe and understand the origin of such emergent phenomena in other layered materials such as twisted hexagonal boron nitride,^37^ and twisted transition metal dichalcogenides, MoS_2_, WSe_2_.^38,39^

**Fig. 1:**
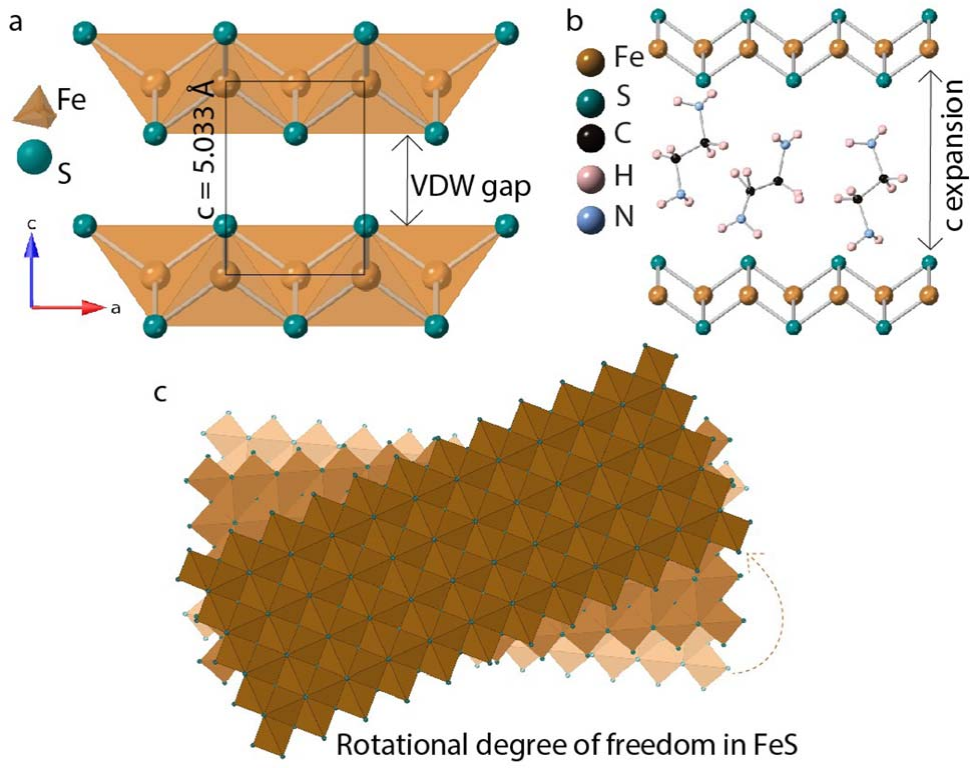
(a) Tetragonal FeS layers (b) guest solvent EDA molecules intercalated in the van der Waals gap (c) twisting of FeS sheets triggered by in-plane rotational degree of freedom.

In this paper, we discuss synthesis and characterization of FeS sheets grown *in situ* under hydrothermal conditions. A reagent excess of ethylenediamine helps with intercalation of neutral amine molecules and tris-ethylenediamine iron(ii) complexes into the layers.^14–16^ FeS layers expand along the stacking direction to accommodate the intercalants (Fig. 1). These guest species can act as structure directing agents and influence stacking orientations of the host FeS sheets powered by hydrogen bonding or other non-covalent interactions. Single crystal diffraction data of iron sulfide intercalated with [Fe(en)_3_]^2+^ previously reported in literature corroborates with the twisting of FeS sheets.^14^

Electron diffraction on the intercalated crystallites show additional Bragg reflections that are not indexed by the parent mackinawite-FeS. The presence of two or more square lattice reflections that appear to be rotated at an angle prompted us to look for formation of Moiré superstructures and the possible relevance of twist angles.^40^ We adopt the coincident site lattice (CSL) theory to explain experimentally observed rotation angle in the diffraction patterns and formation of FeS superstructures.^41^ While CSL theory was initially developed to explain preferred orientations in secondary recrystallization textures in metals,^42,43^ it has been frequently used in other contexts to explain grain boundary dislocations,^44^ orientation relationship between bicrystals^45,46^ and Moiré superlattices.^47–52^ Commensurate superlattices were observed in several homobilayer hexagonal lattices with preference for certain twist angles over other arbitrary angles.^53–56^ This study presents application of CSL theory to interpret high angle twist Moiré superlattices with inherent four-fold rotational symmetry.

## Synthesis and characterization

### Materials and synthesis

For a typical preparation of EDA-intercalated FeS, 4 mmol of Fe powder (Alfa Aesar, 99.5%), 10 mmol of thiourea (Sigma-Aldrich, 99%), 7 mmol of KOH (Fisher, 85%) which is approximately a 0.5 M solution, 10 mL H_2_O, and 3 mL ethylenediamine (Sigma-Aldrich, 99%) sealed within a Teflon cup within a stainless steel autoclave at 120–160 °C for 2–6 days. After the hydrothermal process, the contents were washed and centrifuged with de-ionized water several times until the supernatant was clear. The recovered black powders were collected, vacuum dried and stored in an argon glovebox.

### Material characterization

Powder X-ray diffraction (XRD) data was collected using a Bruker D8 X-ray diffractometer utilizing Cu Kα radiation (*λ* = 1.5418 Å, 2*θ* = 5–70°, step size = 0.020°). Pawley refinements were performed using the TOPAS software.^57^ Microscopic images were examined on a Hitachi SU-70 Field emission scanning electron microscope (FE-SEM), and their elemental compositions were determined by energy dispersive X-ray spectroscopy (EDS) using a Bruker XFlash 6 EDS detector. Electron diffraction patterns were obtained using a JEM 2100 LaB_6_ transmission electron microscope (TEM) at an acceleration voltage of 200 keV. TEM samples were prepared by dispersing the samples in ethanol and drop casting onto a holey carbon film supported on a copper grid.

## Results and discussion

### Hydrothermal ethylenediamine intercalation

Amines can intercalate as both neutral molecules and negatively charged amides or imides. For hydrothermal growths, two main parameters open to change are reaction time and temperature. It should be noted that all successful hydrothermal intercalations of EDA occurred at 120 °C; higher temperatures led to increased unintercalated FeS and Fe_3_O_4_. The results of varying the reaction time are presented in Fig. S1.† For the sulfide analogue, it was found that successful intercalation was observed after two day reaction time; however, some parent FeS remained. At four days, the FeS had been removed and was fully intercalated by EDA. After six days, no intercalation was observed and only poorly crystalline FeS and iron powder remained.

SEM images on the as-synthesized powders show clusters of FeS sheets with lateral dimensions ranging between 5–20 μm (Fig. S2a–c†) with occasionally large flakes of up to 60 μm (Fig. S2d†). Elemental analysis using EDS shown in Fig. S2e† is in general agreement with previous reports for mackinawite,^10,58^ direct stoichiometry cannot be matched due to remaining iron and iron-containing impurities. Elemental analysis for H/C/N cannot be performed with EDS due to out-of-energy resolution (for H), their low collective efficiency (C and N), and highly overlapping spectra of C generated from specimen and carbon tape used for mounting samples. The sample does not appear to be air sensitive after exposure for a week.

### Powder diffraction

Powder X-ray diffraction (PXRD) collected on the powder sample shows few reflections with good signal to noise ratio, a feature commonly observed in layered materials. Pawley fits were used to model the diffraction data since the intercalated structure is too complex to solve by Rietveld analysis with laboratory PXRD. Pawley refinement in tetragonal *P*4 symmetry yielded lattice parameters of *a* = 3.6811(4) Å *c* = 20.6165(1) Å shown in Fig. S3.† This unit cell dimensions are in close agreement with literature reports on alkali metal and alkali metal-free ethylenediamine intercalated FeS.^14,15,24,59^ There is one impurity peak seen in the PXRD due to excess Fe powder remaining from the *in situ* growth of FeS layers. To avoid excess Fe, we attempted syntheses using pre-reacted tetragonal FeS at 120 °C over 2–6 days, but were unsuccessful. Remaining Fe powder could be mostly removed from the recovered product by using a permanent magnet.

Preferred orientation of the 2D sheets allows for better periodicity along stacking direction and stronger 00*l* reflections. Because the layers are held together by weaker van der Waals interactions, disorder in the *ab*-plane is more prevalent which leads to broadening of non-zero *hk*-reflections with low signal to noise ratio (I/I_0_). Because of the limited information from laboratory PXRD, we employ electron diffraction in TEM to extract more structural information from our as-synthesized material.

### Electron diffraction

**Fig. 2:**
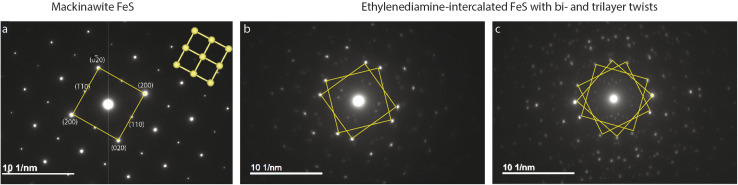
Electron diffraction of (a) unintercalated FeS shows square lattice reflections *vs.* (b) and (c) EDA intercalated FeS. Superlattice reflections corresponding to multiple square lattices seen with constant twist angles.

Plate-like morphology of the FeS crystallites allows them to lay flat on the TEM copper grid, making the [001] zone axis of the crystal parallel to the incident electron beam direction (Fig. S4†). Selected area electron diffraction (SAED) of unintercalated FeS in this direction shows a clear square lattice with predominant {002} and {110} families of reflections (Fig. 2a). We notice square lattice reflections appearing in pairs or triplets of spots, with a constant twist angle (Fig. 2b and c).

To model and simulate the observed ED patterns, we consider two different twist scenarios. The first falls under a bicrystal category and the second is formation of superstructures. The term “bicrystal” refers to two crystals with different orientations sharing a common grain boundary. Meaning, pairs of FeS crystallites grow along the *c*-axis, and these crystals rotate in the ab-plane along the boundary. To simulate ED patterns for a bicrystal, two sets of diffraction patterns of the parent crystal are overlapped and rotated by the measured twist angle. The method is adopted from literature where YBa_2_Cu_3_O_7−*δ*_ (YBCO) thin film bicrystals are rotated by 24°.^60^ The overlapping YBCO grains show Moiré fringes at the grain boundary and the electron diffraction pattern shows symmetric doublets of spots with their angle bisected by mirror planes.

To understand what causes the second set of reflections in Fig. 2b, we first consider possibility of a bicrystal formation. Simulated ED pattern of a mackinawite bicrystal consists of two calculated diffraction patterns of mackinawite rotated by 22.9°. This simulated pattern when superposed on the measured data seen in Fig. S5† revealed several reflections, albeit weak, are not accounted for. Presence of reflections at distances 2.22 and 2.91 nm^−1^ circled in pink are indicative of unit cell larger than mackinawite. The superlattice reflections therefore rule out bicrystal formation leading to the observed diffraction pattern.

**Fig. 3:**
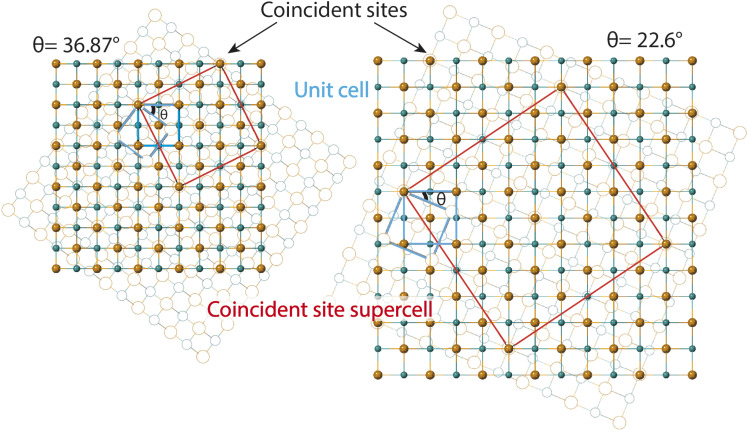
Twisted FeS sheets forming coincident site superlattices (CSLs). The base layer in solid spheres is fixed and the layer in open circles in background is twisted by prescribed angles *θ*. The basic unit cells of mackinawite-FeS are drawn in blue. The coincident site unit cells drawn in red are for twist angles of 36.87° and 22.6°.

The second method we adopt to simulate experimentally observed ED patterns is to build superstructures of mackinawite. The superlattices are composed of atomic positions with perfect overlap or ‘coincident’ sites and will be referred to as coincident site lattices or CSLs. The area and number of iron and sulfur sites included in a CSL depends on the rotation angle. The larger the angle, the smaller the CSL (Fig. 3). CSLs are labelled with a Σ index depending on the periodicity of recurrence of a coincident site *i.e.* a Σ5 CSL implies every fifth site of Fe is coincident (Fig. S7b†). Detailed equations that relate the rotation angle to Σ and the area of a CSL are elaborated in the next section. For the angles that we observed in SAED, we built superstructure models corresponding to Σ5 and Σ13 CSL. Individual superstructures, their electron diffraction analysis, relevance of the measured angles, and how they relate to the coincident site lattice theory are described in the next subsections.

SAED on several other intercalated crystallites is shown in Fig. S6.† These electron diffraction (ED) patterns show features ranging from polycrystalline rings, multitude of twinning artefacts to ordered superstructures. A common trait in most of the ED patterns, however, is the presence of square lattice reflections that are suggestive of the parent tetragonal mackinawite phase. The samples analyzed here are solvothermally grown with no manual control over twist angles or stacking. Under such preparation conditions, several factors can cause inhomogeneity in the layer orientation and crystal growth.

### Misorientation angle and coincident site lattice CSL

Ranganathan has reported in 1966 (ref. 41) that when two identical cubic lattices are rotated, for specific angles, some of the sites in the two lattices overlap and the periodicity of these sites and their length scales are related to the initial lattice by a multiple. The paper put forward a generating function that relates the angle of rotation to Σ, an index that quantifies number of coincident sites and area contained in the supercell formed by rotation. This was later extended to other Bravais lattices by different groups and we refer to CSLs in tetragonal systems by Grimmer in 2003.^61,62^ Grimmer lists Σ and *m* values for rotations by angle *θ* that lead to CSLs but remain independent of the axial *c*/*a* ratio.^62^ The theoretical results from this work show fixed angles result in CSL in tetragonal systems, and we have directly observed these angles in the formation of CSL in our intercalated FeS systems.

The angle of rotation *θ* in terms of rotation axis is described in eqn (1).1
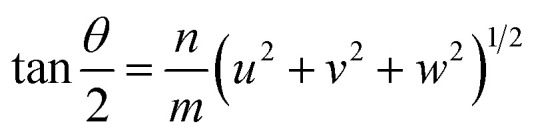
where *m*, *n* are non-zero integers, [*uvw*] is axis of rotation and *n* = greatest common divisor of *u*, *v* and *w*. Since the rotation axis about which the FeS layers are rotated in our case is [001], *n* = 1 and the previous equation can be simplified to:2
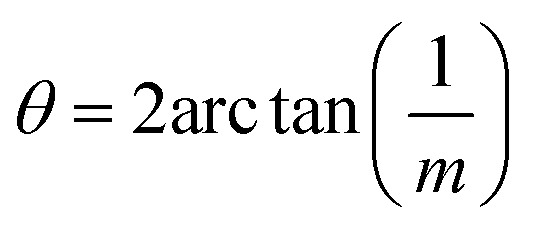


Therefore, an *m* = 1 CLS yields a rotation of *θ* = 90°, which is equivalent to the 4-fold rotation and therefore leads to complete coincidence of all Fe and S sites in first layer to the next. Subsequently, *m* = 3 corresponds to 36.87° and *m* = 5 to 22.62° which lead to partial coincidence shown in Fig. 3. We observe the 22.6° rotation angle directly in our bilayer twist structure and an incommensurate 53.13° rotation in the trilayer twist structure. Although the angle 53.13° is not directly mentioned in the CSL literature, its symmetry equivalent 90–53.13 or 36.87° rotation leads to *Σ*5 CSL in cubic and tetragonal systems.

The next step is to relate *θ* to *Σ*. They are indirectly related through *m*, *n*, [*uvw*] and a proportionality constant *α* shown in eqn (3).3
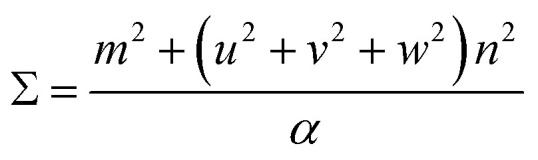


For all common misorientations *Σ* is odd, therefore *α* can only have values 1,2 or 4. Substituting all values same as previous, with additional *α* = 2, *m* = 3 yields *Σ*5 and *m* = 5 yields *Σ*13 respectively. This generating function reported by Ranganathan establishes relation between twist angle and the *Σ* index. *Σ* itself relates the coincident site supercell to the original lattice by quantifying the number of shared sites between the two lattices and the length scale of the new periodicity through eqn (4) and (5).4
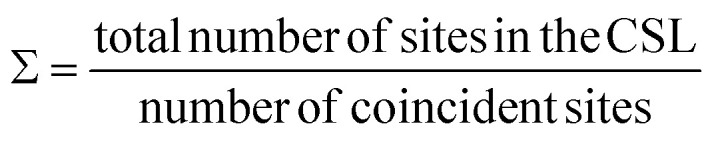

5 *a*′^2^(area of CSL) = *Σ* × *a*^2^ (area of original unit cell)

where *a*′ is the lattice parameter of the CSL and *a* is that of the original lattice.

For the angles observed in our SAED patterns, we deduced values of *m*, *Σ* and area of the supercell using equations described by CSL theory and tabulated below in Table 1. We find excellent agreement between agreement between the observed supercell obtained from SAED and the duduced value from the CSL calculations.

**Table 1:** Misorientation angles observed in SAED for which CSLs exist and relevant calculated values

*θ*	*m*	*Σ*	*S*_CSL_ = Σ × (*a*^2^)	*a*′	*S*_CSL_ = (*a*′^2^)
36.87°	3	5	67.469	8.214	67.491
22.62°	5	13	174.636	13.245	175.477

### Bilayer twist

**Fig. 4:**
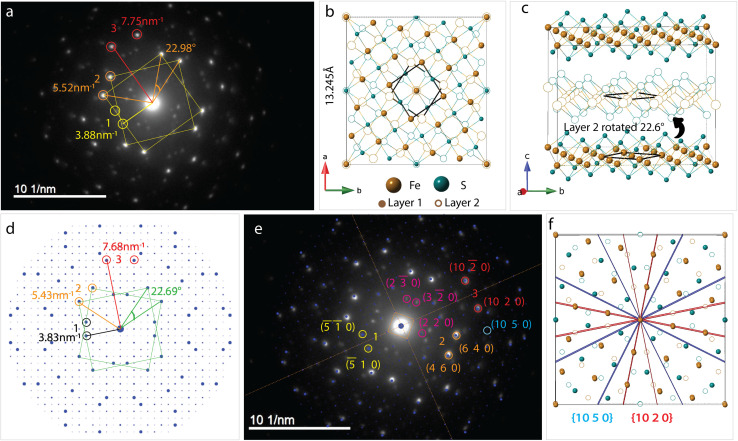
Two layer 22.6° twist (a) measured SAED pattern (b) model of supercell viewed down *c*-axis with second layer rotated 22.6°. The base layer FeS is represented in solid spheres and rotated layer in open circles (c) supercell viewed along *a*-axis (d) simulated ED from supercell model (e) simulated pattern in 4d superposed on 4a (f) families of lattice planes corresponding to reflections circled in red and blue in 4e.

SAED pattern in Fig. 4a shows two sets of square lattice reflections twisted by an angle of 22.98 ± 0.5°. Reciprocal distances of the reflections labelled 1, 2, 3 in the SAED pattern are 3.88, 5.52 and 7.75 nm^−1^. These distances correspond to (110), (200), and (220) planes respectively in mackinawite. CSL theory tells us that twisting square lattices or FeS layers in our case, by 22.6° can lead to a coincident site supercell.^62^ As described by equations in the previous section, twist angle of *θ* = 22.6° leads to an *m* = 5, or Σ13, CSL as shown in Fig. 3b. Each mackinawite unit cell contains 2 unique Fe sites occupying all corners and the face center in the *ab*-plane, located at (0,0,0) and (0.5,0.5,0) (Fig. S7a†). Using the Fe atom at origin as reference, we observe that for 22.6° rotation, every 13th Fe atom in both layers overlap perfectly causing a coincident site (Fig. S7c†). The same goes for sulfur, hence the index *Σ*13. The rotation angle of 22.6° and corresponding coincident index of *Σ*13 obtained by twisting of FeS sheets are in agreement with the CSL theory.

To simulate electron diffraction pattern from the Σ13 CSL, a model has to be built first. For the same twist angle, several new FeS CSLs can be constructed with the coincident sites as corners of the unit cell shown in Fig. S8.† Of the options available, we built second to smallest supercell with 2Fe–2S coincident sites instead of the smallest 1Fe–1S cell. This is to draw parallel with mackinawite that contains 2 iron and sulfur sites and to be consistent with the CSL theory math described previously. Meaning, volume of a *Σ*13 CSL is 13 times that of the initial unit cell, which matches with volume contained in a 2Fe–2S CSL in this case. The new unit cell dimension for a 2Fe–2S CSL is *a*′ = 13.245 Å and its volume is close to 13 times that of a mackinawite cell (Table 1). Details on how the construct the *Σ*13 CSL from mackinawite are mentioned in crystallography section of ESI.† The new model maintains tetragonal symmetry but the twisting of layers to create supercells lowers the symmetry from *P*4/*nmm* in mackinawite-FeS to *P*4/*n* in the *Σ*13 CSL.

Simulated pattern from the *Σ*13 model is shown in Fig. 4d. Brighter reflections appear in pairs similar to the experimentally measured data. Distances of reflections labelled 1–3 and the angles between them are in agreement with the observed values. With the *Σ*13 model we are now able to index reflections circled in pink in Fig. 4b where the bicrystal model failed. All the above mentioned reflections with their corresponding *hkl*-indices are shown in Fig. 4e.

Although we could not extract quantitative information from the SAED intensities, we could note, qualitatively, that the intensities of the experimental and calculated SAED patterns follow the same trend. Calculated patterns, however, have several spots that are not seen in measured data. For example, the (10 2 0) reflection is one of the brighter reflections in the SAED, whereas negligible intensity could be observed for the (10 5 0) reflection. A simple argument of electron density contained in each of the lattice planes can help explain observed intensity at a rudimentary level. Both these reflections are circled in Fig. 4e and drawn out on the model in Fig. 4f. The significant difference in the electron density between (10 2 0) and (10 5 0) planes should lead to the observed intensity although it can be seen in the calculated pattern. The former cuts through iron atom centers whereas the latter does not intersect atomic centers.

Another aspect to address with regard to intensities of reflections in observed and calculated patterns is the simulation settings. Single crystal™ software^63^ was used to simulate all electron diffraction patterns discussed here. Key variables that can be changed in the viewing window are spot size, brightness and gamma correction. Standard software settings initially displayed only the brightest spots with negligible intensity from weaker reflections seen in Fig. S9a.† Although, we noticed that there are several other reflections of negligible intensity being indexed. By increasing the value of gamma, a correction factor in the software that helps accentuate weak reflections that are masked in the presence of very strong reflections, we were able to index all reflections in the experimental electron diffraction pattern. The effect of gamma correction on the simulated pattern and other relevant settings used such as spot size, intensity saturation are presented in Fig. S9.†

### Trilayer twist

**Fig. 5:**
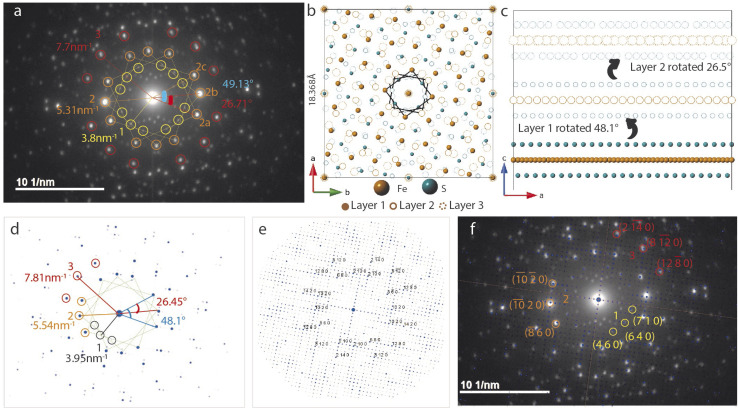
Three layer twist (a) experimental SAED with significant angles, distances labelled (b) supercell model with first layer rotated by 48.1° and second by 26.5° with respect to the base layer is represented in solid spheres (c) supercell viewed along the *b*-axis (d) simulated pattern from the model showing triplets of bright reflections at distances close to what is seen in the measured data (e) simulated pattern with increased gamma correction factor from 1 in 5d to 2 now shows all the weak reflections (f) simulated pattern in 5e superposed on 5a.

SAED in Fig. 5a differs from the bilayer twist (Fig. 4a) with intense reflections occurring in triplets instead of doublets. The distances of reflections labelled 1–3 from the beam center are consistent with the bilayer twist structure, however, the angle between reflections 2a–2b is 26.71° and 2a–2c is 49.13°. While neither of these angles form superlattices according to the CSL theory, incommensurate rotation of the FeS layers could be the reason for the observed superstructure. Since the angles deviate from those described by CSL theory, we refer to this rotation as incommensurate.

These measured angles may seem random at first glance, but we have identified a convoluted relationship with the *Σ*5 CSL formed by 36.87° twist (Fig. 3). Because of the inherent fourfold rotational symmetry, angles *θ* and 90 − *θ* generate identical orientations, making 53.42° and 36.87° equivalent. We consider the possibility of a combination of half and full angle rotations. In other words, reflections 2a and 2c can result from 53.42° twist and reflection 2b due to a third layer that twists by 26.71°.

To build this incommensurate superstructure, we need a triple coincident site between three layers where layer 1 is fixed, layer 2 is rotated by 53.42° and layer 3 by 26.56°. As seen in Fig. S10,† this rotation method now presents near coincidence of all three layers, but 53.42° twist was quite large and deviated a potential coincident site further away from the other two layers. We found 48.13° to result in better coincidence and this explains the observed angle deviation in Fig. 5a. We therefore built a three layer model with layer 1 rotated 48.13° and layer 2 rotated by 26.56°. Images of the model in directions parallel and perpendicular to the *c*-axis are shown in Fig. 5b and c.

The calculated electron diffraction from this model shown in Fig. 5 matches the experimental data closely and can index all the bright reflections. Increasing the gamma correction value from 1 in Fig. 5d to 2 in Fig. 5e shows all the reflections in the reciprocal space and explains presence of several other reflections in the measured data. Working with the layer arrangement in search for this triple coincident site supercell and its best fit structure helped us understand the origin on angles 26.71° and 49.13°.

### Cause for layer twist–hypothesis

Twisting of the FeS sheets during solvothermal growth to form superstructures is evident from our electron diffraction patterns. However, extracting information on arrangement of the ethylenediamine molecules in the interlayer space or its interactions with the host FeS layers is convoluted.

**Fig. 6:**
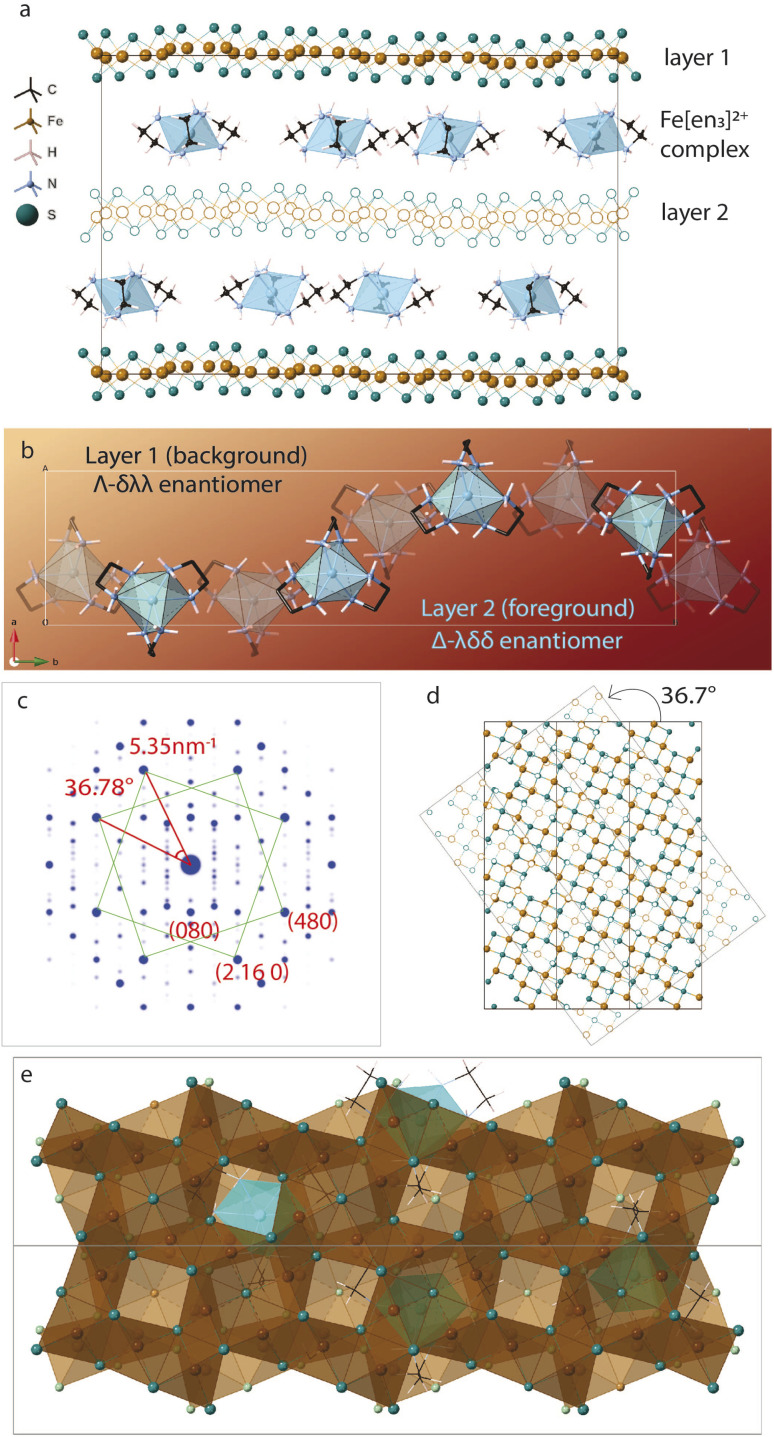
(a) Structure of iron ethylenediamine complex intercalated FeS [Fe_8_S_10_]Fe(en)_3_ en_0.5_ (disordered solvent molecules omitted for clarity) (b) arrangement of the metal amine complexes in between the layers (c) simulated ED pattern of the structure shows two sets of square lattice reflections with twist angle of 36.78° (d) layer 2 rotated 36.7° shows near coincidence with layer 1 displaying a twist orientation relationship between the layers in unit cell (e) layers viewed down the *c*-axis showing Fe vacancies capped by electron density from sulfur sites of next layer or interlayer metal complex.

To understand this, we refer to crystal structure of an analogous compound reported in the literature.^14^ The tetragonal iron sulfide co-intercalated with tris-ethylenediamine iron(ii) complex and uncoordinated ethylenediamine molecules crystallizes in centrosymmetric *P*2_1_/*c* space group seen in Fig. 6a. Individual iron sulfide layers retain the square subnet maintaining semblance to the parent mackinawite. Intercalated structure consists of iron deficiencies and the vacancy ordering in the *ab*-plane leads to an approximate 
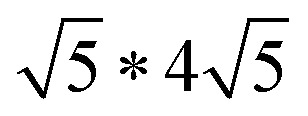
 lattice expansion with *a* = 8.3989(6) Å, *b* = 33.341(3) Å, *c* = 20.551(2) Å, *β* = 90.118(1)°. The vacancies in the FeS layer are created in response to the intercalation of cationic metal amine complexes. To charge balance, the vacancies turn the FeS layers slightly anionic.

As a secondary response to the vacancies, the FeS sheets distort from a perfectly planar structure, lowering in symmetry from tetragonal to monoclinic system with a modulation along the *b*-axis. As can be seen from Fig. 6b, the sinusoidal arrangement of the [Fe(en)_3_]^2+^ complexes in the interlayer galleries also lead to the large *b*–parameter with respect to the *a*–parameter. While the compound contains a racemic mixture of [Fe(en)_3_]^2+^ complexes, which is chiral with D_3_ symmetry, each interlayer gallery contains only one type of enantiomer. Therefore, while the overall structure remains centrosymmetric, locally each layer is chiral.

We observe that the FeS layers are also related by the same misorientation angle as in *Σ*5 CSL, *i.e.* 36.87°. The simulated ED pattern from this crystal seen in Fig. 6c shows similar behavior with pairs of reflections occurring at fixed angle of 36.7°. Concurrently, when layer 2 of the unit cell is rotated in the *ab*-plane, it reaches near coincidence with layer 1 at 36.7° shown in Fig. 6d. While the Fe and S sites coincide with a small offset, some of the vacant Fe sites coincide with vacancy and rest with Fe from the next layer.

Taking a closer look at the crystal structure, it becomes evident that the two FeS layers grow in a staggered conformation. Viewing the layer structure down *c*-axis in Fig. 6e shows the majority of the vacancies in the top layer are either capped by a coordinated sulfur atom from the next layer or an Fe(en)_3_ complex. This staggered configuration allows electronegative sulfur atoms to align with electron-deficient vacancies.

The crystal structure also tells us that the metal complexes within a particular interlayer space are not racemic. Rather, all λ-enantiomers occupy one layer and all Δ-enantiomers in the next, we believe this right and left-handedness from the complex can be translated to the 2-dimensional framework through hydrogen bonds. The positively charged metal amine complexes interact with host FeS layers through electrostatic forces and hydrogen bonding to terminal sulfide anions. N–H⋯S bonds from the chelating amine ligand to adjacent FeS layers vary in bond length from 2.48 Å to 3 Å implying typical hydrogen bond strength. In particular, the intercalated complex is found to have favorable N–H⋯S bonding interactions with the capped sulfur sites.

We hypothesize that the twist orientation may arise from combined effect involving the vacancies created due to the intercalation of positively charged species and non-covalent bonding interactions of the intercalated complexes with the host layers. All these factors, vacancy creation to charge balance positive cation intercalation, staggering of the adjacent FeS sheet to cap the vacancy with electronegative sulfur sites, and N–H⋯S bonding interactions with complex and capped sulfur sites can synergistically contribute toward thermodynamic stability of the twisted conformation.

The crystal structure proves that twisting of layers in van der Waals materials is chemically feasible. While the CSL theory guided us to interpret the twist angles, this structure directed us to look at twisting on an atomic scale. The correlation between twist angles in this structure and our disordered EDA intercalated FeS is not clear, however, it helps gain insight into a potential mechanism of twisting.

### Examples of coincident site lattice in other materials

Ayache *et al.* analysed surface topography and crystallography of the superconducting YBa_2_Cu_3_O_7−*δ*_ (YBCO) bicrystal films on an SrTiO_3_ (STO) substrate.^60^ Their *Σ*13 YBCO bicrystals showed 24° misorientation and the SAED pattern appears similar to what we observed in our *Σ*13 CSL. The difference being in case of YBCO, the observed ED pattern fits well with two rotated diffraction patterns superimposed on each other. Presence of several additional weak reflections that were not reproduced by this superposition method hinted at a superstructure instead of bicrystal.

Wang *et al.* observed only three specific twist grain boundaries associated with *Σ*7, 13, 19 CSLs of the hundred naturally grown Fe_2_O_3_ bicrystals tested.^64^ The CSL orientations in grain boundaries are special because they were found to be lower energy states with additional stability to the boundary gained from stronger interactions through shared sites. Koda *et al.* predicted that 2D crystals preferentially stack with twist angles that would form various CSLs over several other incommensurate misorientations possible.^65^ In 2019, Chen *et al.* reported electrochemical deposition of silver films on silicon wafers.^66^ They observed instead of single unit cell overlap of Ag with Si, a larger CSL with 4 units of Ag and 3 units of Si reduces lattice mismatch from −24.9% to +0.13%.

Zhao *et al.* studied restack behavioral pattern of several TMD bilayers through solution based exfoliation and restacking approach.^54^ The twist angle distribution in homobilayers is not a continuous or smooth pattern; instead, it displays peaks that are determined by short-range commensuration. Interestingly, the most frequently occurring twist angles are found to be ones corresponding to formation of smallest coincidence site lattices.

These are some examples of CSL preferred orientations between two grains in bicrystals or ordered interfaces between 2D crystals or thin film and its substrate or between bilayers. This wide spectrum of examples show that twist phenomena is observed at various length scales from grains to atomic interfaces. CSL theory explains the preference for observed angles is due to formation of low energy grain boundaries or superstructures by sharing of common sites between lattices. As to discussion about our samples, reproducible formation of Fe(en)_3_FeS single crystals with 36.87° rotation between layers is key indicator that the electron diffraction patterns we observed are possible superstructures of mackinawite.

## Conclusion

2D van der Waals layered materials are often vulnerable to turbostratic disorder. In this paper, we present findings of disordered intercalated iron sulfide grown solvothermally in ethylenediamine. The FeS sheets twist *in situ* and the twist angle is not random but can be explained using mathematical concept of coincident site lattices. TEM selected area electron diffraction patterns show multiple superlattice reflections and we have indexed the patterns using two structural models. Bilayer twist discusses FeS superstructure formed due to twisting of two layers by 22.6°. The corresponding supercell is a *Σ*13 CSL with every 13th site being a coincidence site between both layers. Trilayer twist discusses an incommensurate superstructure with three FeS layers per unit cell related to the *Σ*5 CSL. Ideal FeS models were built in both scenarios and respective electron diffraction patterns simulated to prove the formation of these coincident site supercells. The work here primarily focuses on tetragonal iron sulfide, however, the approach and problem solving can be translated to several other layered systems susceptible to similar disorder. Electron diffraction data of intercalated and disorder riddled FeS has not been reported so far and our work addresses this gap in literature.

## Data availability

All experimental details and data supporting the findings of this study are available within the paper and its ESI.† Further data is available from the corresponding authors upon reasonable request.

## Author contributions

Conceptualization: L. B.; B. W.; X. Z.; and E. E. R.; synthesis: H. Z.; electron microscopy measurement: L. B.; B. W.; SC. L.; formal data analysis: L. B.; and E. E. R.; writing and editing: L. B.; and E. E. R.; review and discussion: L. B.; B. W.; X. Z.; SC. L.; and E. E. R; funding acquisition: E. E. R.

## Conflicts of interest

There are no conflicts to declare.

## Supplementary Material

SC-015-D3SC02994H-s001

SC-015-D3SC02994H-s002
