# Supplementary material for: Twisting two-dimensional iron sulfide layers into coincident site superlattices via intercalation chemistry
Source: Chem Sci. 2024 Jan 24;15(9):3223–32. doi: 10.1039/d3sc02994h (PMC10901521; doi:10.1039/d3sc02994h)
Supplement: SC-015-D3SC02994H-s001 [file SC-015-D3SC02994H-s001.pdf]

Electronic Supplementary Material (ESI) for Chemical Science.
This journal is © The Royal Society of Chemistry 2024

Supplemental Information for

# Twisting two-dimensional iron sulfide layers into coincident site superlattices via intercalation chemistry

Lahari Balisetty,[a] Brandon Wilfong,[a,b] Xiuquan Zhou,[a,b] Huafei Zheng,[a] Sz-Chian Liou,[c] and Efrain E. Rodriguez[ab]

[a] Department of Chemistry and Biochemistry, University of Maryland, College Park, MD 20742, USA

[b] Maryland Quantum Materials Center, University of Maryland, College Park, MD 20742, USA

[c] Electron Microscopy Facility, Institute for Functional Materials and Devices, Lehigh University, Bethlehem, PA 18015, USA

E-mail: efrain@umd.edu

**Table of contents**

1. Powder patterns and elemental analysis

2. Electron diffraction analysis

3. Crystallography of twisted layers

   3a. Σ5 CSL

   3b. Σ13 CSL

# 1 Powder patterns and elemental analysis

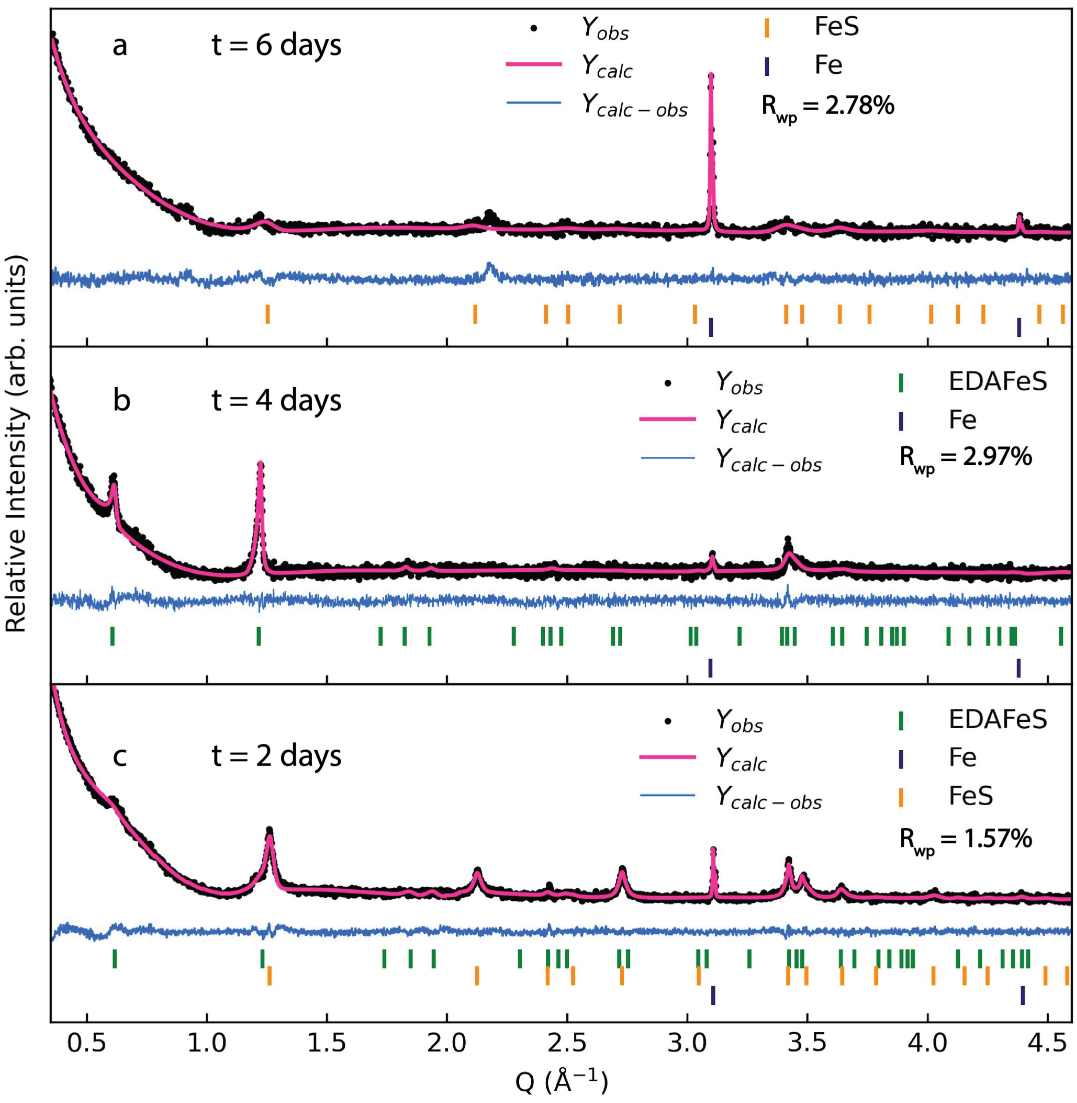


Figure S1: Pawley refinements on ground powders of EDA intercalated FeS after varying hydrothermal reaction times of (a) 6 days, (b) 4 days, and (c) 2 days. EDA intercalated FeS starts to form as a minor phase after 2 days of hydrothermal reaction. As the reaction duration is extended to 4 days, all of the FeS is consumed, and EDA intercalated FeS is the major phase in the reaction medium, followed by the collapse of the intercalated structure when the reaction duration is increased to 6 days.

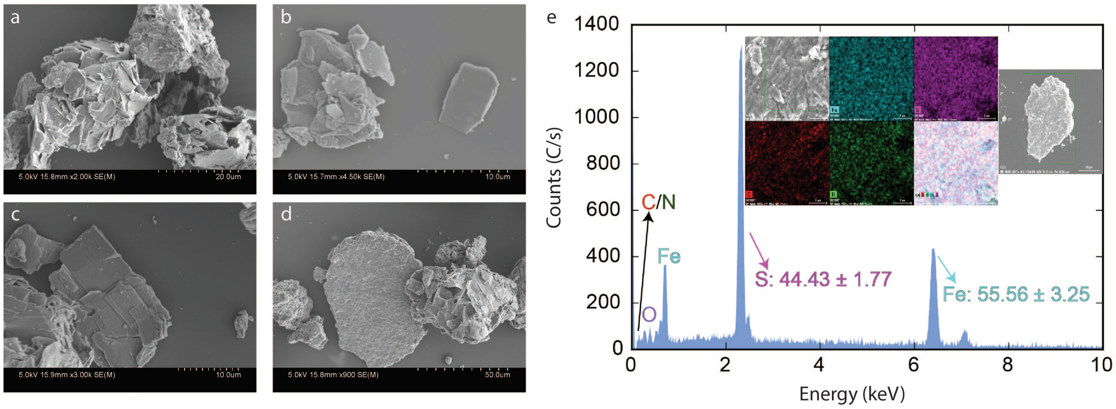


Figure S2: EDS spectrum of EDA intercalated FeS ground powder sample, inset shows SEM image of a typical FeS sheet and its elemental mapping

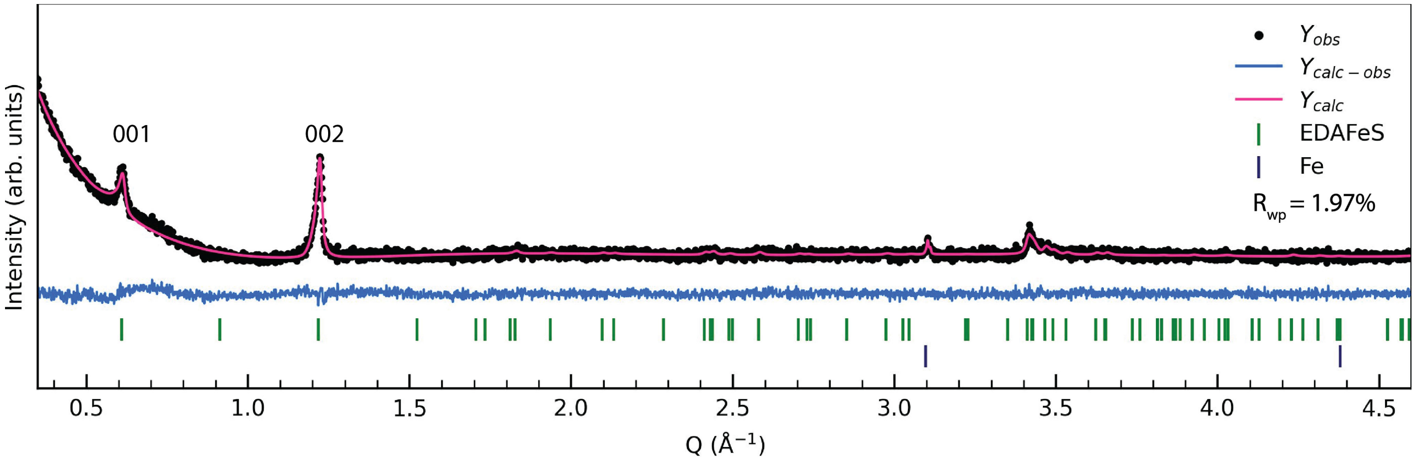


Figure S3: Pawley Fit on EDA intercalated FeS

# 2 Electron diffraction analysis

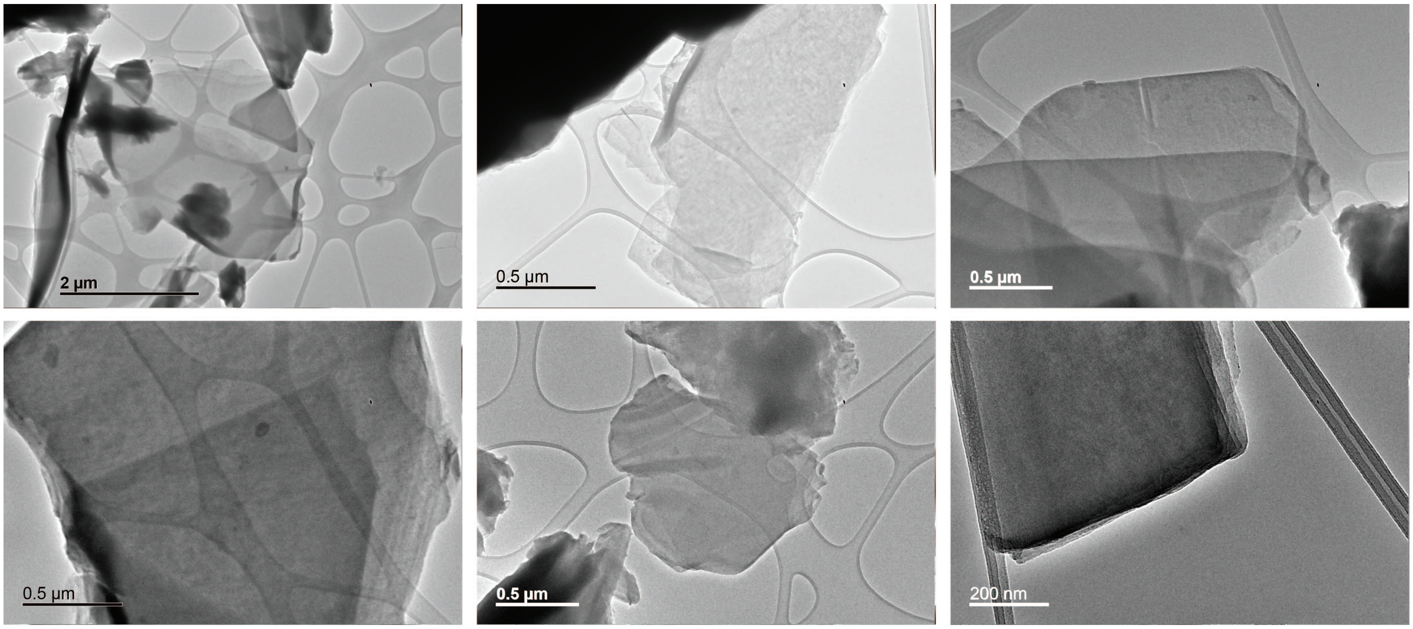


Figure S4: Representative TEM images of transparent FeS sheets

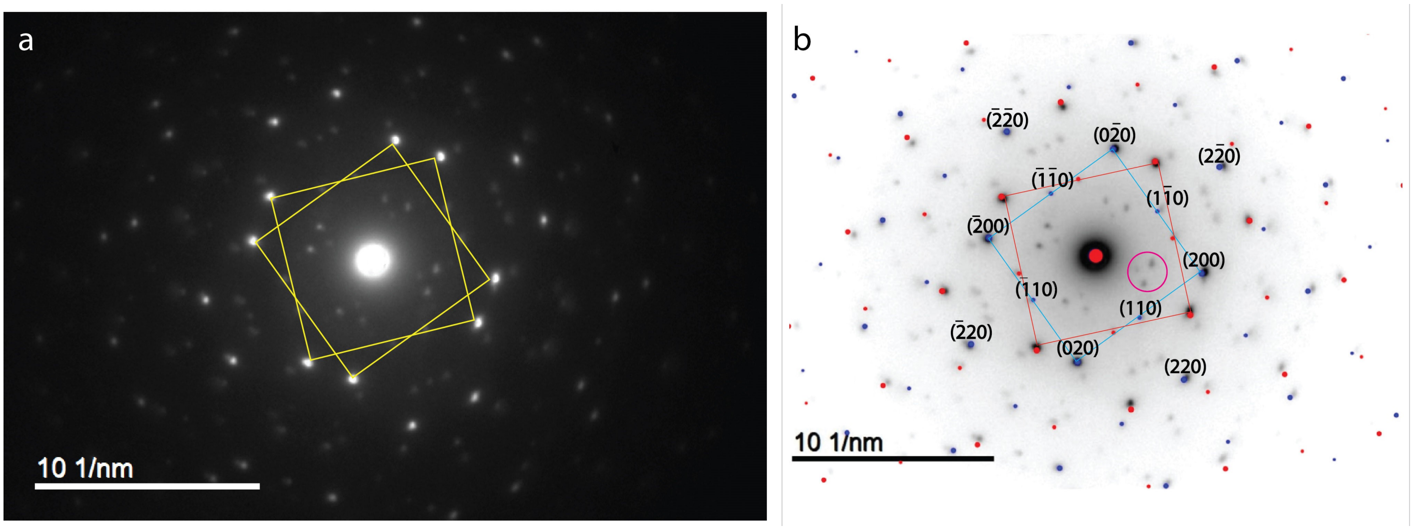


Figure S5: (a) EDA intercalated FeS crystal showing two sets of square lattice reflections (b) Simulated electron diffraction pattern of FeS bicrystal superposed on measured pattern.

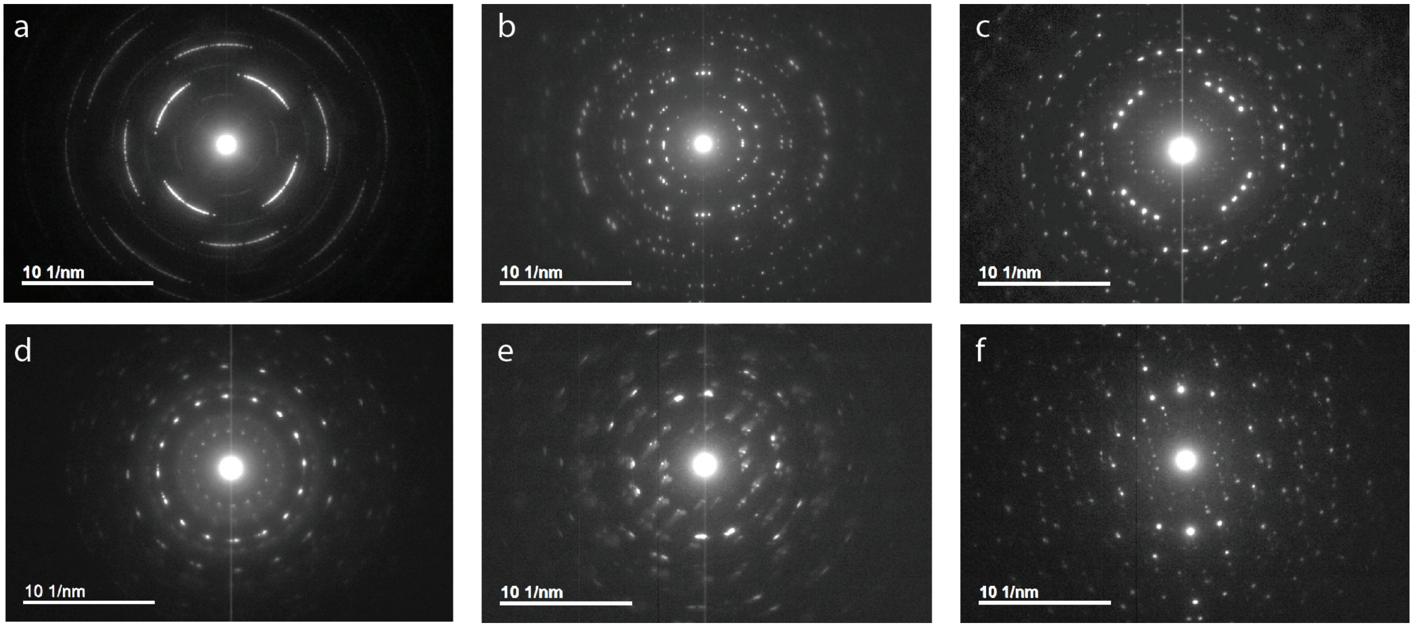


Figure S6: Selected area electron diffraction on various EDA intercalated FeS crystallites showing range of features from polycrystallinity, twinning to ordered superstructures

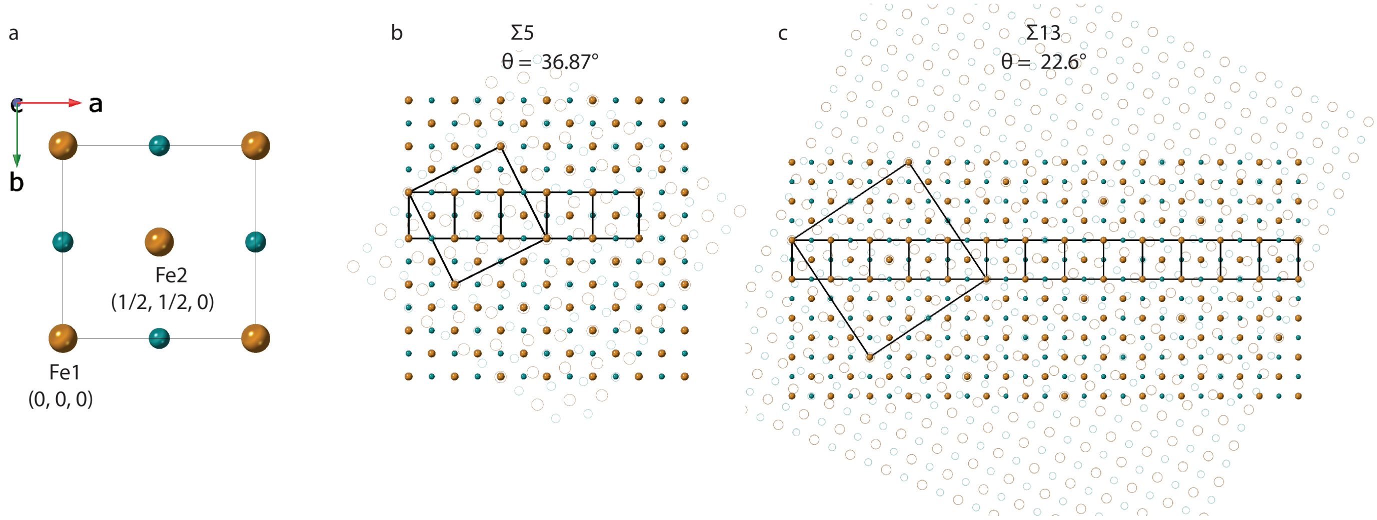


Figure S7: (a) Mackinawite in *ab*-plane with both the Fe sites labelled (b) Frequency of a coincident sites when FeS layers are twisted by 36.87° and (c) 22.6°

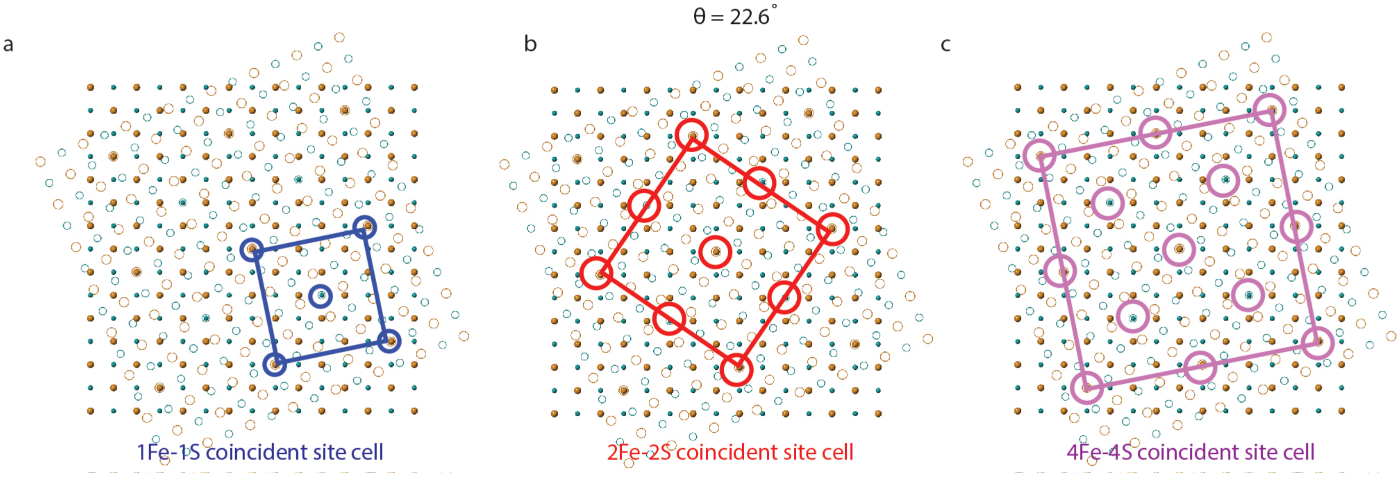


Figure S8: Varying unit volumes of Σ13 CSLs can be built when FeS layers are twisted by 22.6°. Larger the volume, larger the number of coincident sites enclosed (a) 1Fe-1S CSL (b) 2Fe-2S CSL (c) 4Fe-4S CSL

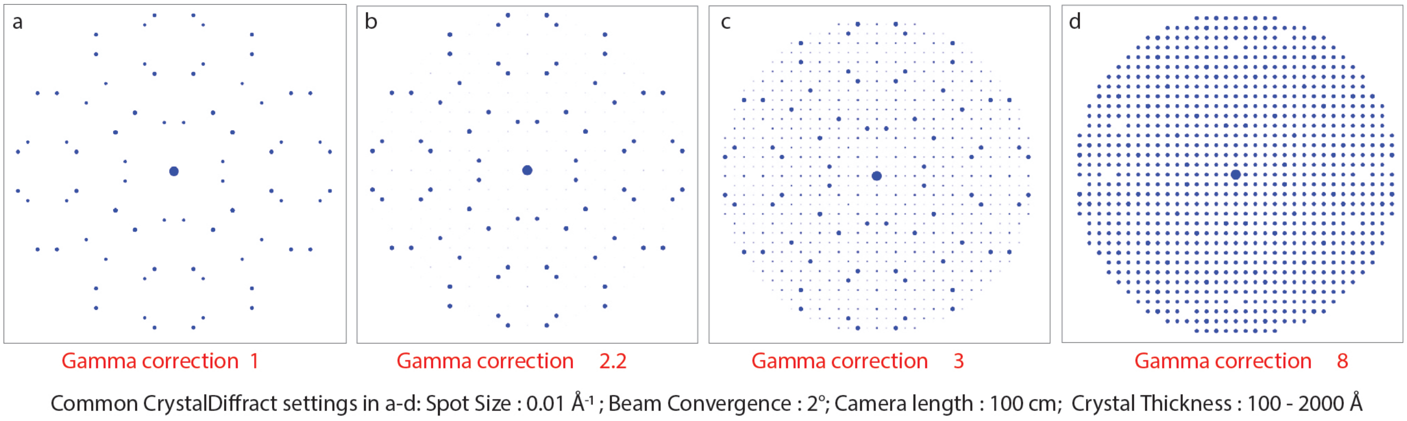


Figure S9: Effect of Gamma correction factor on the simulated electron diffraction pattern. Gamma parameter is an weighted intensity-grayscale mapping function the software CrystalDiffract by CrystalMaker uses to make weaker reflections noticeable in the presence of other bright reflections. Settings in panel (c) were used to index experimental data in the bilayer twist section

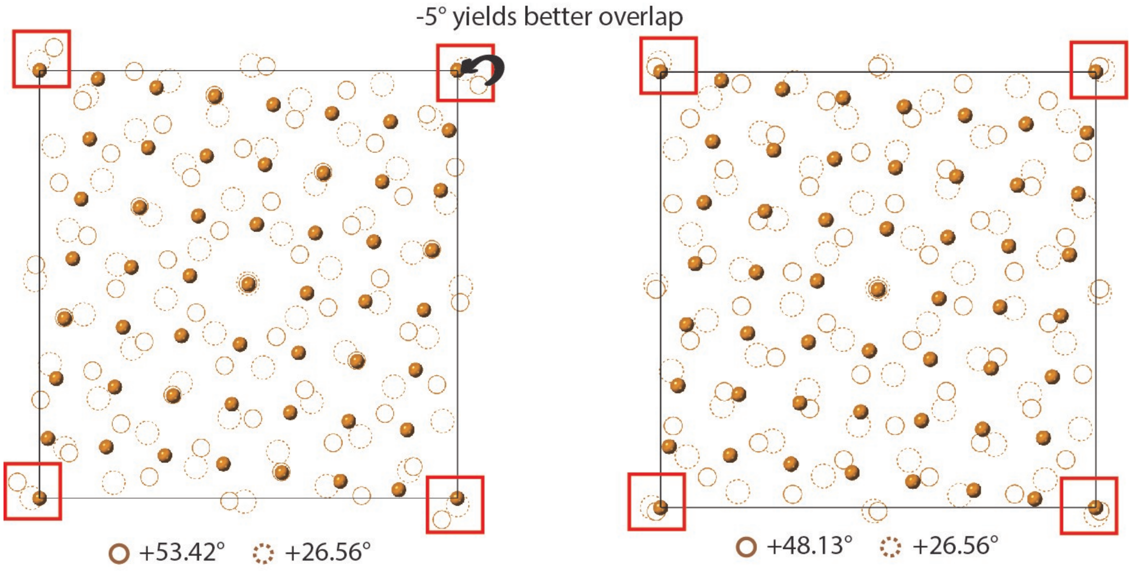


Figure S10: FeS trilayer structure with layer 1 rotated (a) 53.42° vs (b)48.13°. Layer 2 was rotated 26.56° in both cases. Sulfur sites omitted for clarity

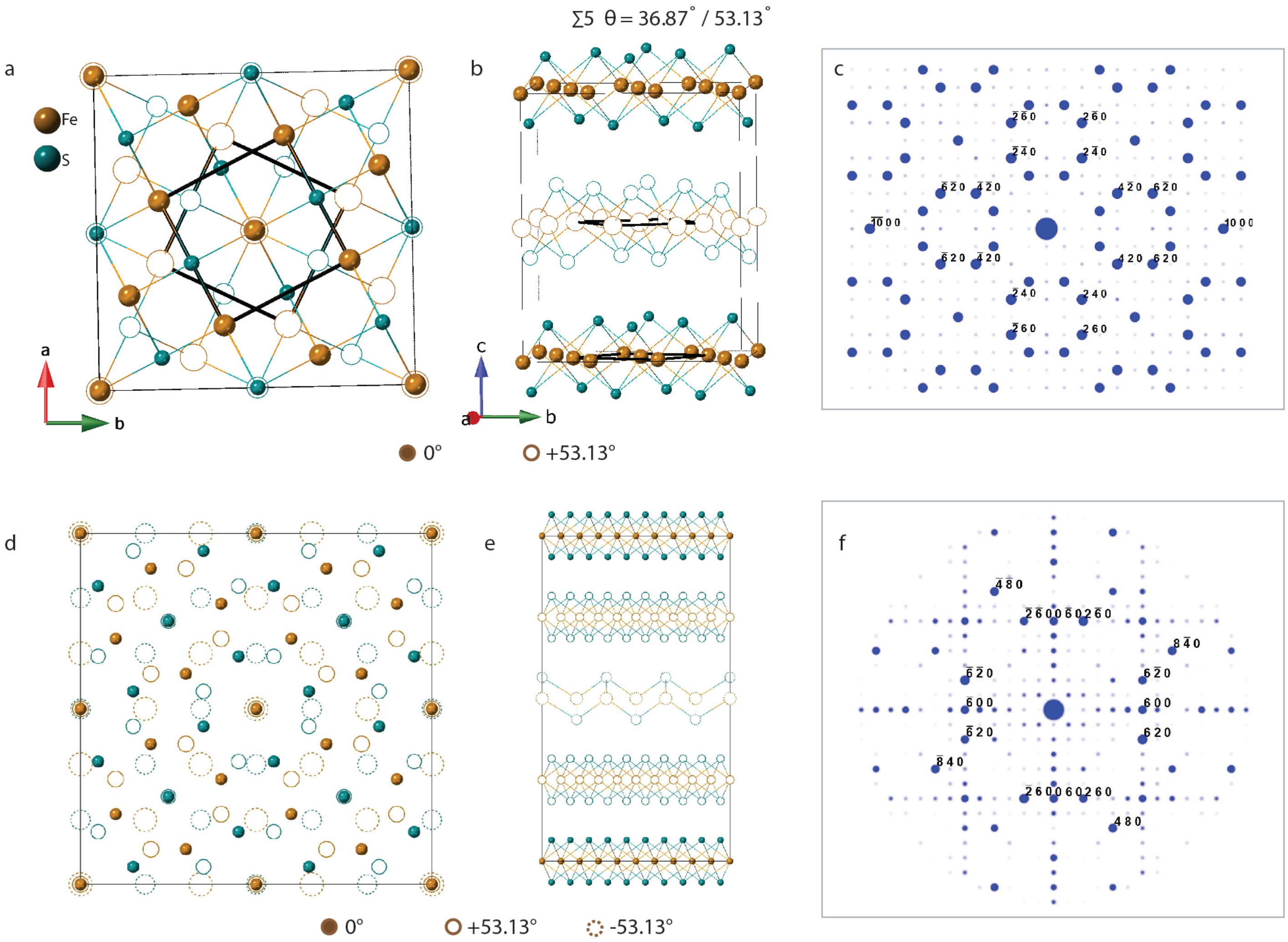


Figure S11: Σ5 CSL. (a,b) Bilayer model viewed along $c$-axis and $a$-axis (c) Simulated electron diffraction from the bilayer model (d,e) Trilayer model viewed along $c$-axis and $a$-axis (f) Simulated electron diffraction from the trilayer model

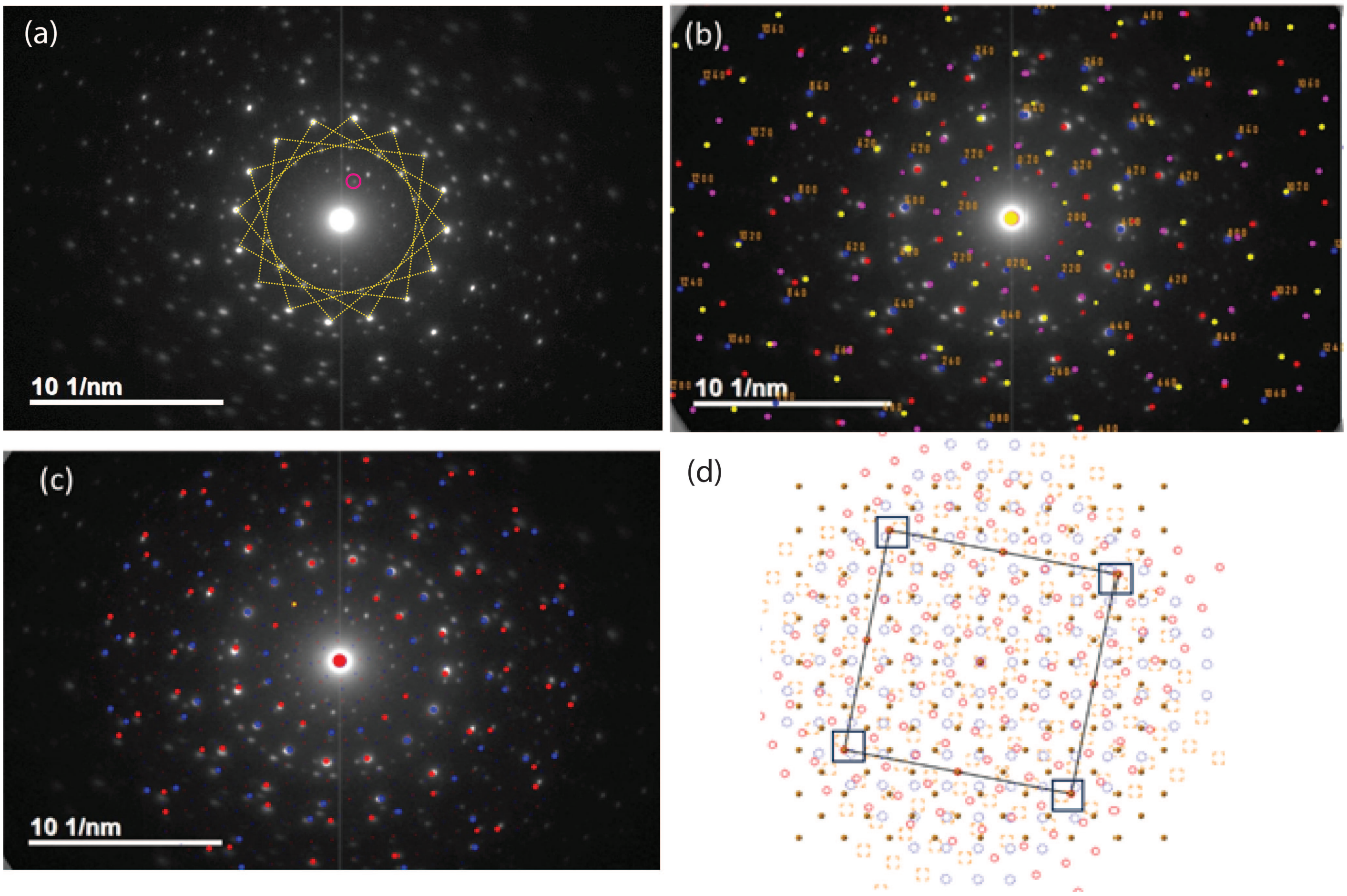

Figure S12: (a) Measured SAED pattern shows symmetric and concentric circles of diffraction spots. Square lattice reflections appear in sets of four (b) Four simulated electron diffraction patterns of 2*2 supercell of mackinawite overlapped with a 22.6° rotation (c) Two simulated electron diffraction patterns of Σ13 CSL overlapped with a 22.6° rotation (d) Twisted layers of FeS (sulfur omitted for clarity). Reflections closest to the beam circled in pink in (a) are not reproduced by simple supercells of FeS. To build a 4layer twist superstructure, we do not observe coincidence site for all four layers away from origin shown in (d). Near coincidence sites are marked at corners of the supercell drawn in black. To build superstructure with these sites coincident requires layers in broken blue circles and broken orange squares to be twisted by 19.6° in clockwise and counterclockwise directions. This deviation is not supported by our data where all measured angles lie within 22.6 ± 1°.

# 3 Crystallography of twisted layers

## 3.1 $\Sigma 5$ CSL

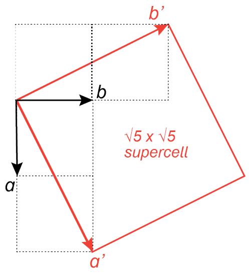


Figure S13: Transformation of the basis set form the smaller tetragonal one to the $\sqrt{5} \times \sqrt{5}$ tetragonal cell. The angle between the two unit cells is 26.56°.

To model the twisting of one square net layer with respect to the other by the angles prescribed in the Common Site Lattice (CSL) method, we begin by describing how the supercell is created by such twisting. We start with the CSL whereby $m = 3$. In this case, the supercell is described by a $\sqrt{5} \times \sqrt{5}$ cell. To transform the basis set from the smaller, original one to rotated supercell, we perform the an operation using the transformation matrix $(\mathbf{P}, \mathbf{p})$ where $\mathbf{P}$ constitutes the linear part of the matrix and $\mathbf{p}$ is a shift vector from $O$ to $O'$. Here, we do not include an origin shift as we keep the $\bar{4}$ operation at the origin for both cells. Figure S13 illustrates how this transformation occurs graphically. Mathematically, we perform the following operation

$$(\mathbf{a}', \mathbf{b}', \mathbf{c}') = (\mathbf{a}, \mathbf{b}, \mathbf{c})\mathbf{P} \tag{S1}$$

$$(\mathbf{a}', \mathbf{b}', \mathbf{c}') = (\mathbf{a}, \mathbf{b}, \mathbf{c}) \begin{pmatrix} 2 & -1 & 0 \\ 1 & 2 & 0 \\ 0 & 0 & 1 \end{pmatrix} \tag{S2}$$

where $(\mathbf{a}', \mathbf{b}', \mathbf{c}')$ correspond to the basis vectors of the new superlattice and $(\mathbf{a}, \mathbf{b}, \mathbf{c})$ those of the original, untwisted lattice. Such a transformation matrix can be used along with the space group $P4/nmm$ (No. 129, origin choice 1), to find the new subgroup to describe the lattice. Using the routine SUBGROUP on the Bilbao Crystallographic Server, the space group with the lowest index ($i = 1$) is $P4/n$ (No. 85, origin choice 1).

To now find the positions of the iron and sulfur atoms in the new superlattice, we must first find the inverse matrix of $\mathbf{P}$. For the case given above, this leads to

$$\mathbf{P^{-1}} = \begin{pmatrix} 2/5 & 1/5 & 0 \\ -1/5 & 2/5 & 0 \\ 0 & 0 & 1 \end{pmatrix} \tag{S3}$$

In general, to find the new positions $(\mathbf{x}')$ of the superstructure from the positions $(\mathbf{x})$ of the original unit cell, we perform the following affine transformation.

$$\begin{pmatrix} x' \\ y' \\ z' \end{pmatrix} = \mathbf{Q} \begin{pmatrix} x \\ y \\ z \end{pmatrix} + \mathbf{q} \tag{S4}$$

where the $\mathbf{Q}$ is equivalent to the inverse matrix $\mathbf{P^{-1}}$ and $\mathbf{q}$ is the column part that captures the shift vector and is given by $\mathbf{q} = -\mathbf{P}^{-1}\mathbf{p}$. Simplifying the transformation, no origin shift is necessary, and $\mathbf{q} = 0$. We therefore carry out the affine transformation, and generate the new positions by the following multiplication

$$\begin{pmatrix} x' \\ y' \\ z' \end{pmatrix} = \begin{pmatrix} 2/5 & 1/5 & 0 \\ -1/5 & 2/5 & 0 \\ 0 & 0 & 1 \end{pmatrix} \begin{pmatrix} x \\ y \\ z \end{pmatrix} \tag{S5}$$

Therefore, to generate the first layer twisted with respect to the $\sqrt{5}\times\sqrt{5}$ cell, the new coordinates, $\mathbf{x}'$, of the iron and sulfur atoms will be given by

$$\begin{aligned} x' &= \frac{2}{5} + \frac{1}{5}y \\ y' &= -\frac{1}{5}x + \frac{2}{5}y \\ z' &= z \end{aligned}$$

Finally, in order to generate the other twisted layer, we simply use the same formulation but now twist the layer in the opposite direction, which leads to the transformation matrix $\mathbf{P}$ being described as

$$(\mathbf{a}', \mathbf{b}', \mathbf{c}') = (\mathbf{a}, \mathbf{b}, \mathbf{c}) \begin{pmatrix} 2 & 1 & 0 \\ -1 & 2 & 0 \\ 0 & 0 & 1 \end{pmatrix} \tag{S6}$$

Since the next layer is translated by half a unit cell in the c-direction, the overall transformation for the coordinates is given by the augmented matrix

$$\begin{pmatrix} x' \\ y' \\ z' \\ \hline 1 \end{pmatrix} = \left(\begin{array}{ccc|c} 2/5 & -1/5 & 0 & 0 \\ 1/5 & 2/5 & 0 & 0 \\ 0 & 0 & 1 & 1/2 \\ \hline 0 & 0 & 0 & 1 \end{array}\right) \begin{pmatrix} x \\ y \\ z \\ \hline 1 \end{pmatrix} \tag{S7}$$

so that the coordinates of the second, twisted layer are given by

$$\begin{aligned} x' &= \frac{2}{5}x - \frac{1}{5}y \\ y' &= \frac{1}{5}x + \frac{2}{5}y \\ z' &= z + \frac{1}{2} \end{aligned}$$

Below a table is created for the positions of both twisted layers given Equations S5 and S7 for the iron positions. It is obvious from the coordinates of the new iron positions that Fe2 and Fe4 in Table 1 the common sites in the lattice. The atoms at the general position (8g) lead to the twisting of the layers at the prescribed angle of 36.87°.

Table 1: Coordinates of of the iron positions $(x, y, z)$ after applying the transformations from Equations S5 and S7 to generate the $m = 3$ CSL. The original positions of the iron are given by $(\frac{1}{2}, \frac{1}{2}, 0)$ and $(0, 0, 0)$. Atoms labeled Fe1 and Fe2 belong to the first twisted layer; Fe3 and Fe4 belong to the second twisted layer. The same procedure can be used to generate the sulfur atom positions. The Wyckoff positions are given for space group $P4/n$ (No. 85, origin choice 1).

| atom | $x'$ | $y'$ | $z'$ | Wyckoff position |
|---|---|---|---|---|
| Fe1 | $\frac{3}{10}$ | $\frac{1}{10}$ | 0 | 8g |
| Fe2 | 0 | 0 | 0 | 2a |
| Fe3 | $\frac{1}{10}$ | $\frac{3}{10}$ | $\frac{1}{2}$ | 8g |
| Fe4 | 0 | 0 | $\frac{1}{2}$ | 2b |

### 3.2 $\Sigma 13$ CSL

To construct the $m = 5$ CSL structure, we use a supercell that is $\sqrt{13} \times \sqrt{13}$. In order to construct the supercell, we use the same method applied for the $m = 3$ CSL structure. Figure S14 illustrates graphically how we built the supercell for one layer. For the first layer we use the following transformation

$$(\mathbf{a}', \mathbf{b}', \mathbf{c}') = (\mathbf{a}, \mathbf{b}, \mathbf{c}) \begin{pmatrix} 3 & -2 & 0 \\ 2 & 3 & 0 \\ 0 & 0 & 1 \end{pmatrix} \tag{S8}$$

which yields the following matrix for the coordinates

$$\begin{pmatrix} x' \\ y' \\ z' \end{pmatrix} = \begin{pmatrix} 3/13 & 2/13 & 0 \\ -2/13 & 3/13 & 0 \\ 0 & 0 & 1 \end{pmatrix} \begin{pmatrix} x \\ y \\ z \end{pmatrix} \tag{S9}$$

For the next layer, which we call Layer 2, we use the following similar set of transformations

$$(\mathbf{a}', \mathbf{b}', \mathbf{c}') = (\mathbf{a}, \mathbf{b}, \mathbf{c}) \begin{pmatrix} 2 & -3 & 0 \\ 3 & 2 & 0 \\ 0 & 0 & 1 \end{pmatrix} \tag{S10}$$

$$\left(\begin{array}{c} x' \\ y' \\ z' \\ \hline 1 \end{array}\right) = \left(\begin{array}{ccc|c} 2/13 & 3/13 & 0 & 0 \\ -3/13 & 2/13 & 0 & 0 \\ 0 & 0 & 1 & \frac{1}{2} \\ \hline 0 & 0 & 0 & 1 \end{array}\right) \left(\begin{array}{c} x \\ y \\ z \\ \hline 1 \end{array}\right) \tag{S11}$$

To generate the new set of coordinates for the iron atoms in the $\sqrt{13} \times \sqrt{13}$ cell, we use the iron atoms at the original coordinates of $(x, y, z) = (0, 0, 0)$. and $(\frac{1}{2}, \frac{1}{2}, 0)$ In addition, since the cell is larger than that of the $m = 3$ supercell, we must include the iron atoms at the corners of the unit cell, so we use the coordinates of $(x, y, z) = (0, 1, 0)$, and $(1, 1, 0)$. To summarize, the new coordinates for iron atoms at both layers 1 and 2 are given in Table 2.

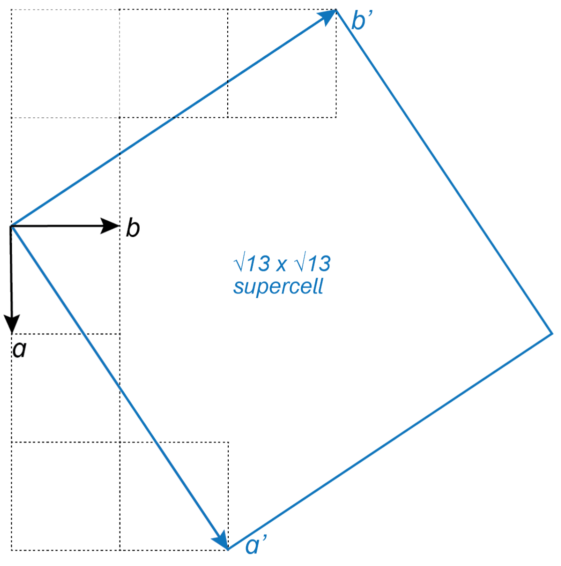


Figure S14: Transformation of the basis set form the smaller tetragonal one to the $\sqrt{13} \times \sqrt{13}$ tetragonal cell. The angle between the two unit cell is 33.69°.

Table 2: Coordinates of of the iron positions $(x, y, z)$ after applying the transformations from Equations S9 and S11 to generate the $m = 5$ CSL. The original positions of the iron were $(0, 0, 0)$, $(\frac{1}{2}, \frac{1}{2}, 0)$, $(1, 1, 0)$, and $(0, 1, 0)$. Atoms labeled Fe1 to and Fe4 belong to the first twisted layer; Fe5 and Fe8 belong to the second twisted layer. The same procedure can be used to generate the sulfur atom positions. Here, Fe1 and Fe5 would constitute the common sites of the CSL. The Wyckoff positions are given for space group $P4/n$ (No. 85, origin choice 1).

| atom | $x'$ | $y'$ | $z'$ | Wyckoff position |
|---|---|---|---|---|
| Fe1 | 0 | 0 | 0 | 2a |
| Fe2 | $\frac{5}{26}$ | $\frac{1}{26}$ | 0 | 8g |
| Fe3 | $\frac{5}{13}$ | $\frac{1}{13}$ | 0 | 8g |
| Fe4 | $\frac{2}{13}$ | $\frac{3}{13}$ | 0 | 8g |
| Fe5 | 0 | 0 | $\frac{1}{2}$ | 2b |
| Fe6 | $\frac{5}{26}$ | $\frac{25}{26}$ | $\frac{1}{2}$ | 8g |
| Fe7 | $\frac{5}{13}$ | $\frac{12}{13}$ | $\frac{1}{2}$ | 8g |
| Fe8 | $\frac{3}{13}$ | $\frac{2}{13}$ | $\frac{1}{2}$ | 8g |
